# Case Report: Benefits of a NSCLC Patient With EGFR A289G/F287_G288insHA cis Mutations From Immunotherapy in Combination With Antiangiogenesis and Chemotherapy and Sequential Treatment of EGFR-TKI

**DOI:** 10.3389/fonc.2022.826938

**Published:** 2022-02-17

**Authors:** He Zhang, Weiwei Dong, Huixia Zhao, Yanyan Hu, Xia You, Tingting Sun, Wenhua Xiao

**Affiliations:** ^1^ Department of Oncology, The 5th Medical Center of Chinese PLA General Hospital, Beijing, China; ^2^ The Medical Department, Jiangsu Simcere Diagnostics Co., Ltd., Nanjing, China; ^3^ Nanjing Simcere Medical Laboratory Science Co., Ltd., Nanjing, China; ^4^ The State Key Lab of Translational Medicine and Innovative Drug Development, Jiangsu Simcere Diagnostics Co., Ltd., Nanjing, China

**Keywords:** nonsmall cell lung cancer (NSCLC), EGFR extracellular domain (ECD) mutations, immunotherapy, antiangiogenesis agents, EGFR-TKI

## Abstract

We presented a 67-year-old nonsmoking female lung adenocarcinoma patient with novel epidermal growth factor receptor (EGFR) A289G/F287_G288insHA cis mutations who responded positively to sintilimab combined with regorafenib and albumin paclitaxel, and sequential treatment of icotinib. Gene mutations in patients were detected by next-generation sequencing (NGS) technology, and changes in gene mutations before and after treatments were observed by ctDNA monitoring. We observed the efficacy of the patient through chest computed tomography (CT) imaging and carcinoembryonic antigen (CEA) level and found that the patient benefited from immunotherapy in combination with antiangiogenesis and chemotherapy for more than 1 year, CEA levels initially fell sharply and then rebounded during the treatment period. After changing to EGFR-TKI therapy, the CEA level of the patient does not only decreased sharply at the initial stage of treatment but also rebounded and increased at the later stage of treatment. The patient was tested for genetic mutations after 4 months of sequential EGFR-TKI therapy and was found to have lost all previous EGFR mutations, which may be the cause of resistance to targeted drug icotinib. We believe that our findings have enriched the EGFR mutation spectrum in NSCLC and highlighted the possible choice for patients harboring this mutation by immunotherapy combined with chemotherapy and antivascular therapy, and EGFR-TKI-targeted therapy.

## Introduction

Owing to favorable and beneficial results from clinical trials, epidermal growth factor receptor tyrosine kinase inhibitors (EGFR-TKIs) have been approved as a standard regimen for patients with nonsmall cell lung cancer (NSCLC) gaining EGFR-sensitive mutations (1). Classical activation mutations (exon 19 deletion and L858R point mutation) account for the vast majority of EGFR mutations and are clearly defined as strong predictors of good clinical response to EGFR-TKIs (2, 3). In addition, low-frequency mutations include point mutations, deletion mutations, insertions, and duplications in exons 18–21 of the EGFR gene in NSCLC and their response to EGFR-TKIs has been studied (4, 5). However, there are a few studies on other regions of EGFR, especially the extracellular region, and there is a lack of effective data on the response of those EGFR variants to targeted drugs.

The therapeutic approach to NSCLC has dramatically shifted with the identification of targetable driver mutations and the introduction of immune-checkpoint inhibitors (ICI) (6). Patients with EGFR-sensitive mutations have limited benefit from immunotherapy, and clinical studies of immunotherapy have generally excluded patients with EGFR-sensitive mutations (7). However, there are also many clinical studies of immunotherapy in patients with EGFR-sensitive mutations. Clinical trial IMPower150 suggested that combination treatment with VEGF inhibitor, checkpoint inhibitor immunotherapy, and platinum-based chemotherapy was effective in EGFR-sensitive mutant nonsquamous nonsmall cell lung cancer (8). Moreover, combination of atezolizumab, bevacizumab, pemetrexed, and carboplatin for metastatic EGFR-mutant NSCLC patients also has good results (9). However, they are all “classical” EGFR mutations, and little is known about the impact of treatment in rare EGFR mutations. More studies about the relationship between rare mutations in EGFR and ICIs benefits are needed. Here, we report an NSCLC patient with EGFR exon 7 F287_G288insHA, as well as a cis Exon7 A289G mutation, who responded positively to sintilimab combined with regorafenib and albumin paclitaxel, and sequential treatment of icotinib.

## Case Presentation

**Figure 1:**
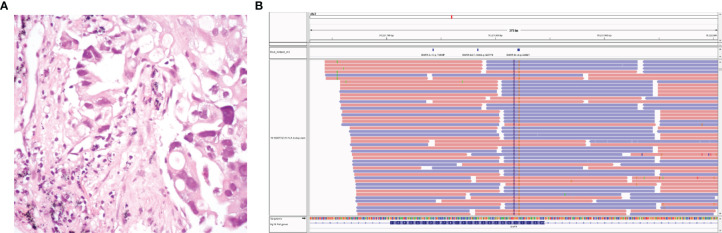
The patient’s pathology revealed lung adenocarcinoma **(A)**. Next-generation sequencing data of circulating tumor DNA indicated two somatic mutations: EGFR p.A289G (c.866C>G) and EGFR p.F287_G288insHA (c.861_862 ins CACGCA) **(B)**.

**Figure 2:**
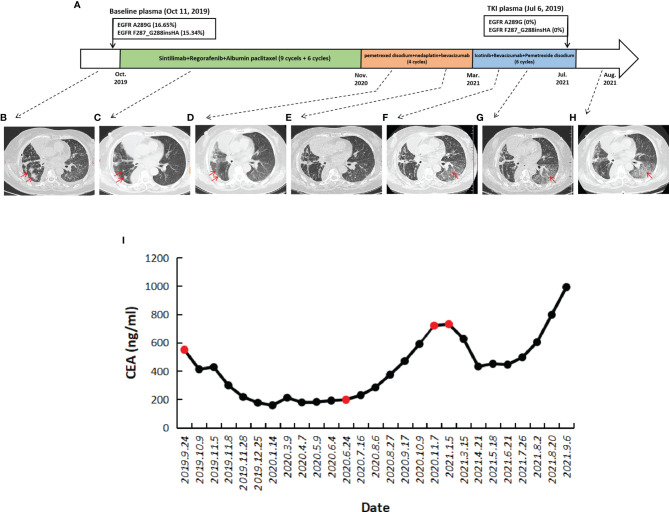
Treatment course and corresponding clinical features of patients after EGFR mutation was detected. **(A)** The patient received immunotherapy in combination with chemotherapy and antiangiogenic therapy, followed by chemotherapy, and finally EGFR-TKI in combination with chemotherapy. From October to December 2019, the treatment effect was the best, and the right lower lung lesion was significantly reduced. **(B)** The CT image of the patient before sintilimab in combination with regorafenib and chemotherapy in October 2019. **(C)** The CT image of the patient after therapy for 2 months in December 2019. CT images from December 23, 2020 **(D)** and February 5, 2021 **(E)** were clinical data of the patient receiving chemotherapy again. Mild interstitial changes in the left lower lung were observed during treatment with EGFR-TKI-targeted drugs icotinib **(F–H)**. **(I)** The variation CEA from the initial treatment to the end of treatment. CEA level decreased sharply after sintilimab in combination with regorafenib and chemotherapy and reached the lowest point in January 2021. After that, it remained stable for a period of time and began to rise in July 2020, and it peaks in January 2021. After the treatment with icotinib in March 2021, the CEA level of the patient decreased significantly, remained stable from April to June, and began to rebound in July, with the CEA gradually increasing. The red dots correspond to the CEA level of the patient before each treatment.

The patient was a 67-year-old nonsmoking woman, and due to chest radiographs during physical examination in August 2014, the right lower lung was found to be occupied, and she underwent surgery at an outside hospital. Postoperative pathology showed that right peripheral moderately differentiated adenocarcinoma of lung lower lobe was mainly an adherent adenocarcinoma (**Figure 1A**). The postoperative stage was T1bN1M0 llA but metastasized into stage IV 1 month after surgery. There were no mutations at exons 18, 19, 20, and 21 of the EGFR. She was admitted to our hospital on October 6, 2014 and had been receiving chemotherapy until 2019. At the end of September 2019, the patient developed intermittent cough accompanied by chest tightness and suffocation, which could not be tolerated. The re-examination found that the right pleural effusion and swelling mark were elevated. Chest CT showed multiple nodules in both lungs, which were increased and enlarged than before. The change of double pneumonia was worse than before. In October 2019, the patient’s plasma sample was subjected to targeted next-generation sequencing (NGS; Nanjing Simcere Technology Inc., Nanjing, Jiangsu, China) for 539 cancer-relevant genes, suggesting that there were mutations in the EGFR 7 exon, which were F287_G288insHA with a frequency of 15.34% and A289G mutation by 16.65% (**Figure 1B**). Since these mutations were not classical activation mutations, and there was no relevant literature report, according to the patient’s wishes, we prioritized immunotherapy combined with antivascular therapy (**Figures 2A**). In detail, the patient was treated with albumin paclitaxel (200 mg/d1), sintilimab (200 mg/d3), 21 days/cycle, combined with regorafenib 9 times, from October 14, 2019 to June 5, 2020. During this period, the chest CT of bilateral miliary nodules and disordered texture were better than before (**Figures 2B, C**), partial response (PR) was elevated, and CEA level decreased significantly. From June 27 to October 9, 2020, six times of maintenance therapy were given with sintilimab combined with regorafenib. CT was still better than before (**Figures 2C, D**), while clinical evaluation of efficacy was SD, the CEA level was slowly elevated, our patient was concerned about this factor, so we considered changing the treatment plan.

**Figure 3:**
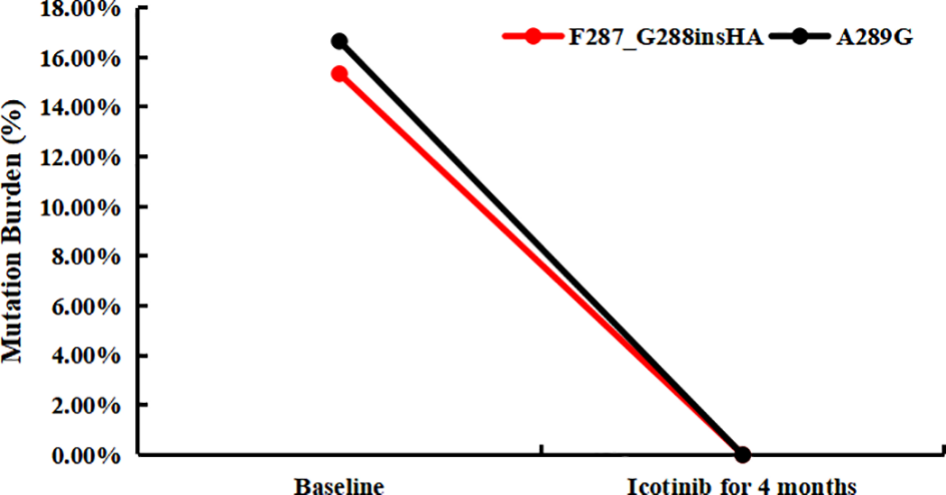
The tumor mutation burden of plasma for novel EGFR mutations in baseline and post-icotinib for 4 months, respectively.

Since the patient had a good response to chemotherapy in the previous treatment, the patient requested to try chemotherapy again. The patient was treated with pemetrexed disodium, nedaplatin combined with bevacizumab 4 times. Chest CT was periodically reviewed to evaluate SD (**Figures 2D, E**), while the CEA level continued to rise. Considering that the patient had EGFR A289G mutation, it was reported that EGFR A289V mutation was sensitive to icotinib in NSCLC (10), so we tried EGFR-TKI-targeted therapy. The treatment was adjusted to pemetrexed disodium and bevacizumab combined with icotinib for 6 cycles. Grade II gastrointestinal reaction, especially diarrhea occurred after taking icotinib. The symptoms were relieved after treatment by regulating intestinal flora. The patient’s condition remained stable (**Figures 2F–H**), and CEA decreased slowly in the early stage, and then increased slowly again 4 months after combination with icotinib. The variation of CEA levels is shown in **Figure 2I** for each visit from the initial treatment to the present. Although the patient’s clinical efficacy assessment was SD, the CEA level of the patient increased significantly, and we also considered whether there was drug resistance, so repeating NGS with plasma was developed on July 5, 2021. Compared with the baseline, the mutations in the abundance of novel EGFR exon 7 mutations for post-icotinib disappeared (**Figure 3**), which may be the reason of icotinib resistance.

## Discussion

Although EGFR mutations have been recognized for a long time and occurred at a frequency of approximately 50% in Asia NSCLC (11), especially “classical” EGFR mutations such as exon 19 deletion and L858R mutations which predicted sensitivity to EGFR-TKIs (12), rare mutations besides exons 18–21 of the EGFR gene have not been enough studied in clinical practice.

The p.F287_G288insHA and p.A289G (c.866C>T) mutations locate on exon 7 of the EGFR gene and encode amino acid of the extracellular domain of EGFR protein. To date, none of them have been reported yet. However, the most common EGFR variant in glioblastoma (GBM) is a deletion from exons 2–7, EGFRvIII, extracellular domain (ECD) missense mutations comprise 10%–15% of transcripts (13), which is constitutively active due to an inframe truncation within the extracellular ligand-binding domain (14). Point mutations in the extracellular region of EGFR such as R108K, A289V/D/T, G598D, and other extracellular domain mutations are identified in 24% of GBM samples (15, 16). These mutations keep EGFR in an active conformation (17).

In addition to TKIs, monoclonal antibodies (mAbs) that competitively bind extracellular receptor binding regions also inhibit kinase downstream signaling pathways. Cetuximab (18) and panitumumab (19) are anti-EGFR mAbs which were approved by FDA to treat patients with metastatic colorectal cancer (mCRC). Cetuximab alone or combined with EGFR-TKIs had been studied in patients with advanced NSCLC, and the higher response rates were seen on cetuximab in randomized trials (20), and dual EGFR blockade with afatinib and cetuximab seemed to induce tumor responses in patients with EGFR exon 20 insertion mutant NSCLC (21). However, resistance of anti-EGFR mAbs was often driven by acquisition of mutations in the ECD of EGFR (22). Cetuximab interacts exclusively with domain III of ECD of EGFR (23); it cannot be sure whether the EGFR p.F287_G288insHA and p.A289G cis mutations would affect protein structure. Therefore, whether patients with this mutation will be sensitive to anti-EGFR mAbs deserves further investigation.

To our knowledge, this is the first report of p.F287_G288insHA and p.A289G cis mutations in NSCLC, and its corresponding treatment with the PD-1 mAb (sintilimab) plus antivascular agents (regorafenib) for more than 1 year, and first-generation EGFR-TKI (icotinib) for more than 4 months. This finding may expand the EGFR mutation spectrum for using ICIs and TKIs as a treatment in NSCLC.

In summary, our case report expands the spectrum of EGFR mutations, in particular, the types of EGFR “variant III.” Furthermore, this case showed a possible choice for patients with this mutation in NSCLC by immunotherapy combined with chemotherapy combined with antivascular therapy or TKIs.

## Data Availability Statement

The original contributions presented in the study are included in the article/supplementary material. Further inquiries can be directed to the corresponding author.

## Ethics Statement

The studies involving human participants were reviewed and approved by The 5th Medical Center of Chinese PLA General Hospital. The patients/participants provided their written informed consent to participate in this study.

## Author Contributions

HZhang and WD conceived the idea. WD is the patient’s primary oncologist. TS and XY drafted the initial draft alongside comprehensive literature review. Additional details are provided by HZhao. YH assisted with material and data collection. WX revised the draft before an additional round of revisions by all authors. All authors listed have made a substantial, direct, and intellectual contribution to the work and approved it for publication.

## Conflict of Interest

Authors XY and TS are employed by Jiangsu Simcere Diagnostics Co., Ltd.

The remaining authors declare that the research was conducted in the absence of any commercial or financial relationships that could be construed as a potential conflict of interest.

## Publisher’s Note

All claims expressed in this article are solely those of the authors and do not necessarily represent those of their affiliated organizations, or those of the publisher, the editors and the reviewers. Any product that may be evaluated in this article, or claim that may be made by its manufacturer, is not guaranteed or endorsed by the publisher.
